# Alarmerend beeld onder personeel ggz; uitputting, angst- en somberheidsklachten met mogelijk vertrek uit de ggz als gevolg: Wat kan de ggz doen?

**DOI:** 10.1007/s12508-022-00353-y

**Published:** 2022-06-24

**Authors:** Anneloes van den Broek, Arno van Dam, Inge Bongers, Yvonne Stikkelbroek, Nathan Bachrach, Lars de Vroege

**Affiliations:** 1 GGz Breburg, Breda, Nederland; 2 Departement Tranzo, TSB, Tilburg Universiteit, Tilburg, Nederland; 3 GGZ WNB, Bergen op Zoom, Nederland; 4 GGZ Eindhoven, Eindhoven, Nederland; 5 GGZ Oost Brabant, Boekel, Nederland; 6 Child Adolescent Studies, Utrecht University, Utrecht, Nederland; 7 Afdeling medische en klinische psychologie, Tilburg Universiteit, Tilburg, Nederland; 8 RINO Zuid, Eindhoven, Nederland

**Keywords:** mentale gezondheid, medewerkers, geestelijke gezondheidszorg, COVID-19-pandemie, Mental health, Employees, Mental health care, COVID-19 pandemic

## Abstract

Nu de COVID-19-pandemie steeds meer geïntegreerd raakt in het dagelijks leven en het aantal ziekenhuisopnamen afneemt, krijgen we meer zicht op de mentale gevolgen van de pandemie voor zorgprofessionals in de geestelijke gezondheidszorg (ggz). Van de ondervraagde ggz-medewerkers (*N* = 1.372) ervaart 50% meer stressgerelateerde klachten en vertoont 30% somberheidsklachten. Tegelijkertijd melden zich meer cliënten met klachten als direct en indirect gevolg van de COVID-19-pandemie bij de ggz. Dit zorgt voor extra werkdruk in een sector waar de wachtlijsten toch al lang waren. Om uitval en/of vertrek van zorgprofessionals uit de ggz te voorkomen is het nodig om te leren omgaan met de consequenties van de pandemie in de spreekkamer en om met preventiemaatregelen stress/burn-out te voorkomen.

**Digitaal aanvullende content:**

De online versie van dit artikel (10.1007/s12508-022-00353-y) bevat aanvullend materiaal, toegankelijk voor daartoe geautoriseerde gebruikers.

## Abstract (en)

While the COVID-19 pandemic integrates more and more into daily life and hospitalizations decrease, the consequence for mental health care personnel is becoming clearer. 50% of the employees in mental health care institutes experience stress and 30% have signs of depression. Simultaneously more patients present themselves at the mental health care institutes with complaints as a result of the COVID-19 pandemic. This increases workload even more while the waiting lists are already very long. To prevent sick leave and/or even resigning, social support in the working environment, prevention measures for mental complaints, and support have to be initiated by every level of the mental health care institutes.

## Inleiding

In het begin van de COVID-19-pandemie werden alle zeilen bijgezet ten behoeve van het welzijn van ziekenhuispersoneel. Zij moesten immers dagelijks aanhoudend een zware inzet leveren. Op deze schaal was dit nog niet eerder aan de orde geweest. Terwijl de COVID-19-pandemie in diverse varianten voortduurt, komen problemen naar voren waar eerder nog geen aandacht voor was, zoals die in de geestelijke gezondheidszorg (ggz). De mentale gezondheid van werknemers binnen de ggz verslechterde als gevolg van de pandemie [1]. De door de regering opgelegde maatregelen om de pandemie en verdere besmettingen te reguleren hebben daarbij dan ook verstrekkende gevolgen voor dit werkveld. Denk bijvoorbeeld aan isolatie/eenzaamheid, disregulatie van werkzaamheden en uitval. Tevens werden spannings-, angst- en somberheidsklachten gerapporteerd, die het werken in de ggz belastender maakten, ook al werden de maatregelen gevoegelijk afgeschaft. Deze bijdrage brengt de gevolgen van de door de COVID-19-pandemie geïnduceerde ontwikkelingen voor medewerkers in de ggz in beeld en vraagt om aandacht voor de situatie en ondersteuning van de medewerkers in deze sector.

Recentelijk gaf de World Health Organisation (WHO) al aan zich zorgen te maken over zorghulpverleners ten tijde van de COVID-19-pandemie. In een rapport merkte de WHO op zich ernstig zorgen te maken over mentale klachten onder hulpverleners, zoals mentale vermoeidheid en overspannenheid [2]. Onlangs werd in Nederland de DFY-study (Don’t Forget Yourself-study) uitgezet onder ggz-medewerkers (in zowel de specialistische als de basis-ggz) [3]. Een zelfrapportagelijst werd verspreid via social media (LinkedIn) en via bestuurders van grote ggz-instellingen in Nederland. Hier zijn in totaal 1.372 reacties op gekomen vanuit ggz-medewerkers van meer dan twintig instellingen verspreid door het hele land. Dit onderzoek toonde aan dat het eerder geschetste beeld van verslechterde mentale gezondheid onder ziekenhuispersoneel in de ggz-sector ook speelt en er tevens sprake is van een hoge werklast en druk [1, 2].

## Toename van mentale klachten

Een substantieel deel van de ggz-medewerkers gaf aan klachten ten gevolge van de COVID-19-pandemie te ervaren [3]. Vijftig procent had meer last van stress/spanning dan voor de COVID-19-pandemie. Dertig procent sprak van een toename van gevoelens van depressie/somberheid en gaf aan dat dergelijke zelfgerapporteerde mentale klachten vooral door de pandemie werden veroorzaakt [3]. De verslechtering van bestaande klachten werd eveneens toegeschreven aan de pandemie. De balans tussen werk en privé zou niet per definitie doorgeslagen zijn naar werk, maar toch vermeldde een deel van de medewerkers dit wel zo te ervaren (gedeeltelijk mee eens 24,6%; mee eens 9,4%), waardoor de druk toenam. Over het algemeen hadden de medewerkers niet meer ziekteverlof en/of meer (losse) vrije dagen opgenomen. Op de vraag of ze het werk anders zouden willen inrichten naar aanleiding van de COVID-19-pandemie gaf 31,5% aan meer thuis te willen blijven/gaan werken, zou 7,5% minder uur willen werken en overwoog 4,2% van de ondervraagden om te stoppen met werken in de zorg. Dit laatste kan een behoorlijke impact hebben, aangezien de ggz reeds voor de pandemie kampte met capaciteitsproblemen. In de digitale aanvullende content van dit artikel zijn de resultaten van de survey in detail terug te vinden.

Onderzoeken in andere landen laten een soortgelijk beeld zien van substantiële stress-, angst- en depressieklachten onder personeel dat werkzaam is in de gezondheidszorg [4]. Uit een onlangs in de *Lancet* gepubliceerd onderzoek bleek dat de prevalentiecijfers van depressie en angststoornissen door de COVID-19-pandemie in zijn algeheel zijn gestegen, vooral onder vrouwen [5]. Deze algehele stijging valt ook te generaliseren naar de ggz-medewerkers – sterker nog, er werken meer vrouwen dan mannen in de ggz, wat maakt dat eerdergenoemde onderzoeksresultaten zorgelijk zijn voor de ggz-sector.

Niet alleen de toename van de mentale klachten onder het ggz-personeel maakt de situatie binnen de ggz precair. Er is daarnaast namelijk door de instroom van patiënten met COVID-19-gerelateerde klachten sprake van een toename van de ggz-wachtlijsten [6]. Deze stijgende zorgvraag betreft individuen die klachten hebben door de COVID-19-pandemie en de gestelde maatregelen (bijvoorbeeld angst- of somberheidsklachten) of (blijvende) klachten hebben na besmetting met COVID-19 en die doorverwezen worden naar de (specialistische) ggz. Een deel van hen heeft daarbij professionele ondersteuning nodig [6]. Na het doorlopen van revalidatieprogramma’s na besmetting met COVID-19, waarin vooral gefocust wordt op fysieke klachten, is er vaak pas later aandacht voor het mentale/psychische aspect van COVID-19. Wanneer de fysieke klachten niet overgaan of niet meer verminderen volgt het acceptatieproces over het voortbestaan van deze klachten. Hierbij moet nieuw copinggedrag worden aangeleerd om de ontwikkelde comorbide mentale klachten te kunnen hanteren. De klachten van individuen met blijvende fysieke klachten na COVID-19 worden veelal geclassificeerd als somatisch-symptoomstoornis, omdat dergelijke klachten doorgaans langer dan 6 maanden aanhouden. Deze patiënten worden hiervoor vaak verwezen naar de specialistische ggz (sggz), waardoor de druk op de sggz verder toeneemt (door een toestroom in reeds bestaande wachtlijsten).

Naast COVID-19-gerelateerde mentale klachten namen depressieve klachten in de bevolking toe als gevolg van eenzaamheid en het wegvallen van activiteiten die vanwege de ingestelde maatregelen niet meer mogelijk waren. De behandelopties voor depressie als gevolg van door de maatregelen vergrote eenzaamheid en vermindering van activiteiten waren door de coronamaatregelen beperkter, wat bij hulpverleners tot gevoelens van onmacht leidde (denk hierbij aan een dagstructuur die vervaagde door de quarantainemaatregelen of de onmogelijkheid om groepen te draaien). Daarbij zorgden de maatregelen ook voor een toenemende agitatie, binnen en buiten de ggz, waarover ook in de behandelkamer gesprekken werden gevoerd (bijvoorbeeld over de mondkapjesplicht). Het anti-overheidsdenken en wantrouwen jegens de overheid en instituties zijn voor sommige cliënten een belangrijk deel van hun leven en lijdensdruk geworden, waarover ze met hun hulpverlener willen praten. Dit stelt eisen aan de hulpverlener, die een balans moet zien te bewaren tussen begrip voor de emoties van de cliënt en de eigen opvattingen over de maatregelen. Dit geldt ook, in toenemende mate, wat betreft het complotdenken rond de COVID-19-pandemie. Het vraagt van de hulpverlener dat deze onderscheid kan maken tussen waanstoornissen, complotdenken en andere meningen, en op deze verschillende fenomenen adequaat kan reageren. Dit zijn vaardigheden waarin niet iedere ggz-medewerker geschoold is [7].

De ggz kampt momenteel met lange wachtlijsten [8]. Dit maakt dat de toename van cliënten op deze wachtlijsten door COVID-19-gerelateerde psychische klachten het werk binnen de ggz extra onder spanning zet. Het tekort aan medewerkers binnen de ggz is slechts een van de redenen van de lange wachtlijsten. Uit onderzoeken die tijdens andere pandemieën verricht zijn, is gebleken dat negatieve emoties gerelateerd zijn aan verminderde werkprestaties en uiteindelijk kunnen leiden tot ontslag [9]. Gerapporteerd werd dat de toegenomen inzet van digitaal werken het werkplezier verminderde. Daarbij komt zoals gezegd dat 4,2% van de zorgprofessionals in de ggz overweegt te stoppen met werken in de ggz [3]. Deze ontwikkelingen tezamen zorgen voor een mogelijke overload aan ggz-behandelaanvragen, een nog grotere toename van de wachtlijst, met mogelijk uitval van collega’s door ziekte en burn-out tot gevolg.

Burn-outklachten kunnen in de huidige situatie op twee verschillen manieren ontstaan. Ten eerste door de ontwikkeling en voortduring van stress als gevolg van de COVID-19-pandemie op zich. Op de tweede plaats door toenemende stress als gevolg van de stijgende instroom van cliënten vanwege de COVID-19-pandemie, met een mogelijk vertrek van collega’s tot gevolg. Het is te verwachten dat hierdoor de toch al hoge werklast en -druk binnen de ggz verder zullen oplopen [4, 10]. De toename van de belasting zet een vicieuze cirkel in gang van verdere verslechtering van de mentale gezondheid onder ggz-personeel en een stijging van het verloop. Eenzelfde ontwikkeling zien we op dit moment bij het ziekenhuispersoneel. De grotere instroom van patiënten in combinatie met een hoog ziekteverzuim, burn-out en uitstroom van (bijvoorbeeld intensive care-)personeel maakt dat de ziekenhuiszorg onder druk komt te staan. Zo’n scenario, waarbij kwaliteitsverlies gevreesd wordt, staat de ggz ook te wachten en/of is reeds sluimerend aan de gang, waarbij vertrek van medewerkers in de ggz een reële consequentie kan zijn. Ter illustratie: op 20 maart 2022 betrof het ziekteverzuim in een aantal grote ggz-instellingen gemiddeld 16%.

## Vergroten van de veerkracht

Het bieden van hulp en ondersteuning aan medewerkers kan erger voorkomen [11]. Uit eerdere onderzoeken die verricht zijn ten tijde van de SARS-pandemie is gebleken dat psychologische ondersteuning effectief is, zowel organisatiebreed als op individueel niveau [12]. Denk hierbij aan het bevorderen van sociale ondersteuning buiten de werkomgeving (door publieke opinie, praktische ondersteuning van familie en vrienden) en het onderhouden van sociale contacten (binnen de mogelijkheden van de gestelde maatregelen). Aanvullend is psycho-educatie over mentale zelfzorg belangrijk, net als preventiemaatregelen (monitoring van het mentaal welzijn en het inbouwen van ontspanning op de werkvloer) om emotionele stress te ondervangen, waardoor uitval op werk voorkomen kan worden [10]. Daarbij is het ook aan te raden om in te steken op zelfmanagement (gericht op het monitoren van de eigen balans), wat binnen de instelling zelf gerealiseerd kan worden door bijvoorbeeld een buddysysteem te creëren of een preventieaanbod door en voor collega’s (teamleden onderling of via een supportafdeling mentaal welzijn). Zo kunnen medewerkers tijdig ondersteund worden door professionals op het gebied van ervaren klachten als machteloosheid, angst- en/of andere mentale klachten. Het is ook essentieel om stigmatisering bespreekbaar te maken, zodat voor collega’s de drempel verlaagd wordt om bij elkaar aan te kloppen voor hulp. Hierbij valt ook te denken aan trainingen die werknemers handvatten bieden en waarbij ze leren om verzet tegen COVID-19-maatregelen en complotdenken bespreekbaar te maken [7]. Het creëren van dergelijke vormen van ondersteuning in een veilige werkomgeving behoeft duidelijke organisatorische strategieën op elk niveau in de instelling.

Zoals eerder gezegd ervaart een groot gedeelte van de zorgprofessionals mentale klachten. Het is interessant om te kijken naar de professionals die deze niet in dezelfde mate ervaren. Deze groep wordt gekenmerkt door een hoge mate van veerkracht. De veerkracht van een organisatie wordt grotendeels bepaald door de veerkracht van haar personeel. Dat betekent dat zorginstellingen hun verantwoordelijkheid moeten nemen om hun medewerkers te beschermen en weerbaar te maken in tijden van een pandemie [10]. Het Nederlands Instituut van Psychologen gaf ook al aan om juist in te zetten op de mentale veerkracht van professionals door in hen te blijven investeren (via opleiding en het creëren van social support op de werkvloer) en in te zetten op preventie, zoals ook het Deltaplan Mentale Vooruitgang stelt [13]. Ook het Trimbos-instituut geeft aan dat de kans op burn-out onder (jonge) zorgprofessionals reëel is en biedt in de leidraad ‘Mentale klachten voorkomen bij zorgmedewerkers’ handvatten voor het uitzetten van beleid dat de veerkracht van zorgmedewerkers verder kan vergroten.

## Conclusie en aanbevelingen

Uit de eerdergenoemde survey blijkt dat de mentale klachten onder medewerkers in de ggz substantieel zijn. Na de COVID-19-pandemie zijn gevoelens van stress en somberheid/depressie onder ggz-medewerkers verder toegenomen, respectievelijk bij 50% en 30% van de ondervraagden in een recente survey. Verder bleek dat 4,2% van de ondervraagden uit deze survey overweegt om volledig te stoppen met werken in de zorg. De ggz kampt met lange wachtlijsten en tegelijkertijd heeft de pandemie mentale consequenties die nog langdurig aanhouden en een grote flexibiliteit vereisen. Cliënten met blijvende klachten door de COVID-19-pandemie of besmetting met COVID-19 vinden meer en meer de weg naar de ggz. Dit geldt ook voor cliënten die mentale klachten ontwikkelen door uitgestelde somatische zorg en de daarmee gepaard gaande beperkingen. Dit maakt dat de capaciteit verder onder druk komen staan, net als de ggz-werknemers door de toename van de werkdruk en -last. Deze ontwikkelingen maken het noodzakelijk om de noodklok te luiden voor de ggz.

Instellingen moeten actief ondersteuning aan het eigen personeel bieden bij mentale klachten en de ervaren werkdruk als gevolg van de COVID-19-pandemie. Diverse wetenschappelijke onderzoeken ten tijde van andere pandemieën benadrukken en onderschrijven het belang hiervan. Het Trimbos-instituut heeft recentelijk een leidraad gepubliceerd die handvatten biedt voor een praktijkgerichte aanpak van mentale problematiek op de werkvloer [14]. Gezien en gehoord worden blijkt daarbij belangrijk te zijn. Het is essentieel dat de signalen op de werkvloer worden opgevangen, zodat werknemers gehoord worden wanneer er problemen ontstaan. Daarbij is het ook van belang dat medewerkers mee kunnen beslissen. De leidraad raadt ook aan om regelmatig navraag te doen naar de gezondheid van medewerkers. Een andere praktische oplossing vormt een inloopspreekuur bij een mental coach. Ook het aanbieden van cursussen/trainingen waarin mentale klachten en de invloed op werkdruk centraal staan zou belangrijk zijn. Een preventieve cursus om overspannenheid te voorkomen kan eveneens soelaas bieden.

Het is niet alleen van belang om de aanwezige klachten daadkrachtig aan te pakken, maar ook om te kijken naar de inrichting van het werk en preventie van de ontwikkeling of verslechtering van mentale klachten. Het bovenstaande betreft keuzen op instellingsniveau, maar die zouden ook moeten terugkomen in het overheidsbeleid. Onzes inziens moeten ze aan bod komen binnen het programma Pandemische paraatheid van het ministerie van Volksgezondheid, Welzijn en Sport. Dat moet ervoor zorgen dat er bij de bestrijding van een nieuwe pandemie sneller en adequater wordt opgestart, en dat er een duurzame aanpak wordt ontwikkeld waarbij zorgprofessionals in tijden van crisis adequaat kunnen inspringen. Het lijkt ons een logische keuze om de ggz bij deze plannen te betrekken. Collega’s onderschreven deze gedachte – zij hebben op social media massaal positief gereageerd op de eerder genoemde onderzoeksbevindingen en een oproep tot participatie van ggz-professionals in het overheidsplan pandemische paraatheid. (We ontvingen maar liefst 150.000 reacties.) Ondersteuning van personeel in de ggz dient gewaarborgd te worden, om zo (verdere) escalatie en de negatieve spiraal te voorkomen die we in het ziekenhuis bij het (intensive care-)personeel zagen.
