# Supplementary material for: Alarming situation amongst mental health care employees; mental health issues with possible resignation as a consequence: What can be done?
Source: TSG. 2022 Jun 24;100(3):107–11. [Article in Dutch] doi: 10.1007/s12508-022-00353-y (PMC9243997; doi:10.1007/s12508-022-00353-y)
Supplement: Supplementary file 1 [file 12508_2022_353_MOESM1_ESM.pdf]

Factsheet uitkomsten Mentale gezondheid

Algemene uitkomsten ten tijde van COVID-pandemie

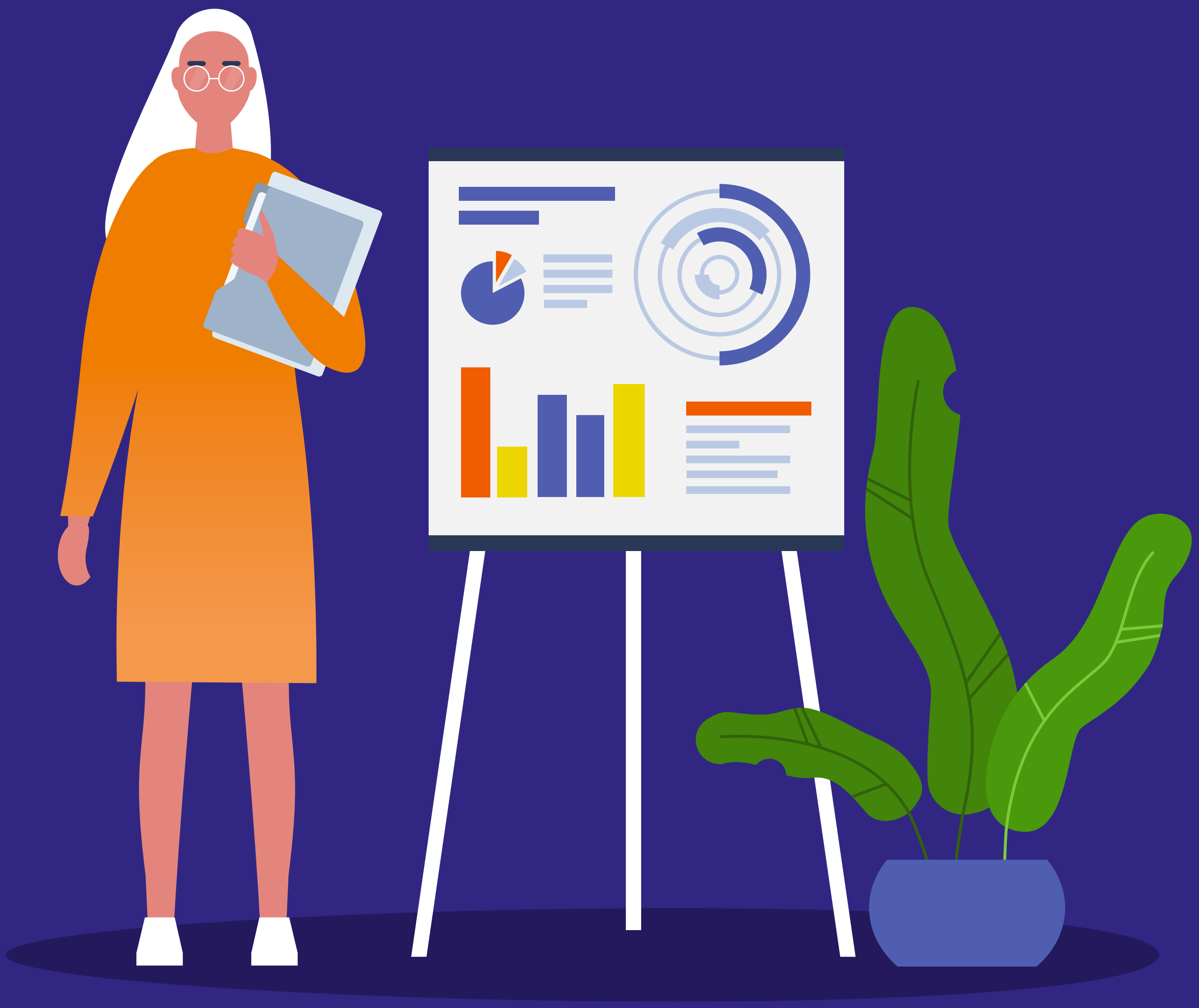

GGz Breburg

De algemente uitkomsten

Gespannenheid

Meer last	48.5%	(n =658)
Niet meer/niet minder last	47.9%	(n = 650)
Minder last	3.6%	(n = 49)

Somberheid

Meer last	30.3%	(n = 409)
Niet meer/niet minder last	67.5%	(n = 911)
Minder last	2.1%	(n = 29)

Angst

Meer last	19.4%	(n = 262)
Niet meer/niet minder last	78.3%	(n = 1055)
Minder last	2.3%	(n = 31)

Verder gaf 23.6% (n = 318) van de respondenten aan meer last te ervaren van gevoelens van boosheid en 14.2% (n = 190) gaf aan meer last te hebben van verdriet. Gemiddeld bezien gaven respondenten aan significant minder goed in hun vel te zitten tijdens de pandemie dan ten opzichte van voor de pandemie.

Over alle respondenten bezien geeft 27.8% aan dat de pandemie enigszins heeft bijgedragen aan dit verminderd functioneren en 28.6% gaf aan dat dit volledig als gevolg van de pandemie gezien wordt.

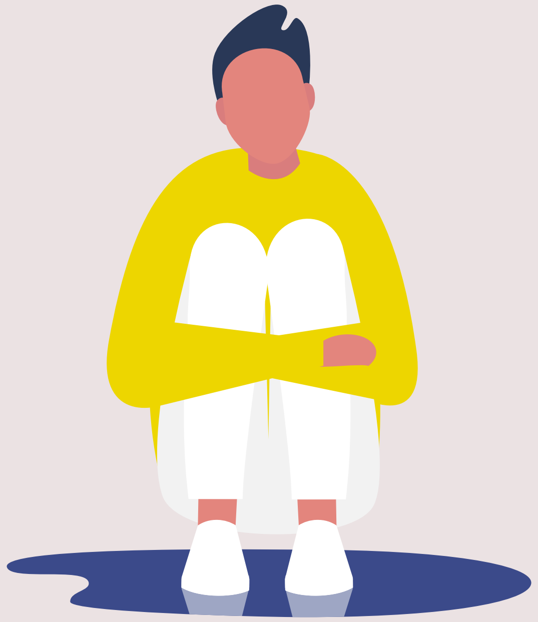

De balans tussen werk/privé is vanwege het thuiswerken meer doorgeslagen naar werk

Mee eens	9.4%	(n = 126)
Gedeeltelijk mee eens	24.6%	(n = 331)
Neutraal	28.8%	(n = 387)
Gedeeltelijk oneens	11.7%	(n = 157)
Oneens	25.6%	(n = 345)

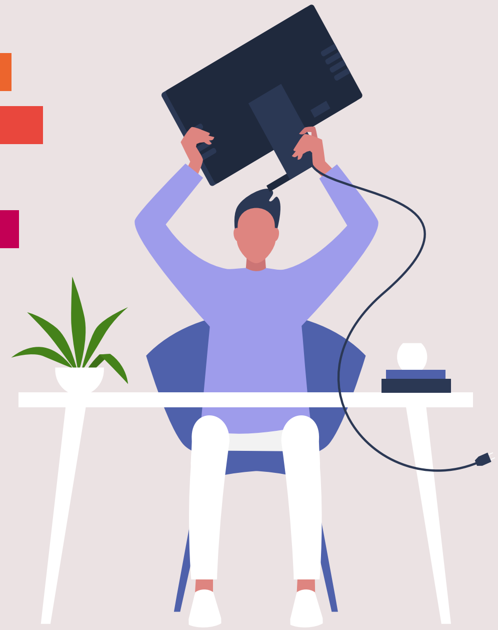

Verder gaf 15.8 tot 33.8% van de respondenten aan minder goed de balans te kunnen houden tussen werk en privé. Over het algemeen bezien gaf men wel aan de balans tussen werk en privé weer terug te kunnen brengen (33.8%). 3.7-16.7% van de respondenten gaf aan dat het hen niet lukt om deze balans (weer) terug te brengen.

Ziekteverlof/verzuim

Ik heb meer ziekteverlof opgenomen

Oneens	68.3%	(n = 925)
Gedeeltelijk oneens	3.5%	(n = 47)
Neutraal	8.1%	(n= 109)
Gedeeltelijke eens	9.0%	(n = 122)
Eens	11.4%	(n = 154)

Ziekteverlof/verzuim

Ik heb meer (losse) vrije dagen opgenomen

Oneens	64.8%	(n = 875)
Gedeeltelijk oneens	5.2%	(n = 70)
Neutraal	10.1%	(n= 137)
Gedeeltelijke eens	12.4%	(n = 168)
Eens	7.5%	(n = 101)

Werk anders inrichten naar aanleiding van de coronapandemie?

Op de laatste vraag, of men naar aanleiding van de coronapandemie overweegt om het werk anders in te richten, gaf 56.9% (n=767) aan dit niet te overwegen. 425 respondenten (31.5%) gaf aan meer thuis te willen blijven/gaan werken, 56 respondenten (4.2%) gaf aan te overwegen om te stoppen met werken in de zorg en 101 (7.5%) van de respondenten gaf aan minder uur te willen gaan werken.

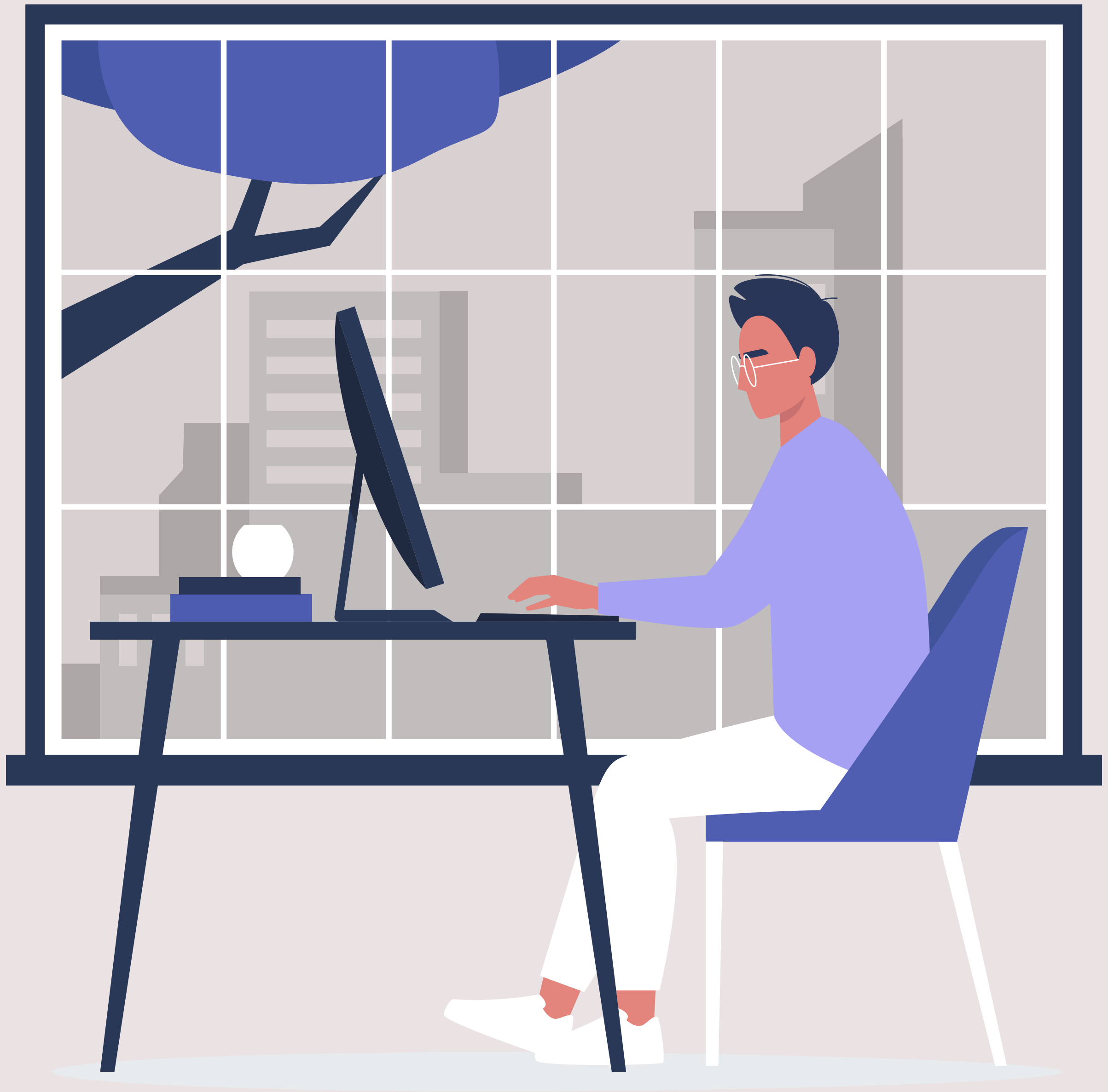

Conclusie

Nu de pandemie ogenschijnlijk zijn plek heeft gekregen in de samenleving worden de mentale gevolgen voor de geestelijke gezondheid nu pas duidelijk. Vanuit deze survey blijkt dat een groot gedeelte van de medewerkers in de ggz 16-55% meer gespannenheid, en gevoelens van somberheid en angst ervaren. Dit zijn substantieële cijfers welke zeer relevant zijn en welke aandacht vragen. Daarbij de verzuimpercentages onder medewerkers mee lijken te vallen en zij aangaven niet minder vrije dagen te hebben opgenomen. De kans op het ontwikkelen van burnout moet gezien deze gegevens niet onderschat worden en is een serieus te nemen signaal. Dit vraagt vanuit ggz-instellingen aandacht en stappen om de mentale gezondheid van hun personeel te ondersteunen. Te meer ook omdat 3-6% van de ondervraagden overweegt te stoppen met het werken in de zorg en de krapte op de arbeidsmarkt binnen de ggz reeds zorgwekkend is. De corona-pandemie heeft dus grote invloed gehad op mentale gezondheid van medewerkers.

Voor meer informatie en indien je alle gegevens wil ontvangen kun je contact opnemen met:

dr. A. (Anneloes) van den Broek (a.vandenbroek@ggzbreburg.nl) en/of
dr. L. (Lars) de Vroege (l.devroege@ggzbreburg.nl).

“We beloven iedereen die een beroep op ons doet, dat we samen de weg zoeken, vanuit alle complexiteit, naar een situatie die je weer aan kunt. Van te groot naar klein genoeg. Zodat jij je leven de baas blijft”

Contact

Tel.: 088 - 016 16 16

info@ggzbreburg.nl

www.ggzbreburg.nl

GGz Breburg
